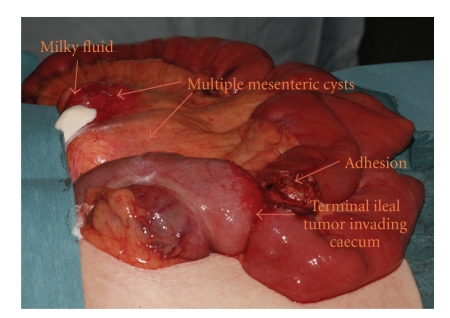# Cavitating Mesenteric Lymph Node Syndrome in Association with Coeliac Disease and Enteropathy Associated T-Cell Lymphoma: A Case Report and Review of the Literature

**DOI:** 10.1155/2010/478269

**Published:** 2011-01-02

**Authors:** Olivia M. B. McBride, Richard J. E. Skipworth, Derek Leitch, Satheesh Yalamarthi

**Affiliations:** ^1^Department of General Surgery, Queen Margaret Hospital, Whitefield Road, Dunfermline KY12 0SU, UK; ^2^Department of Pathology, Queen Margaret Hospital, Whitefield Road, Dunfermline KY12 0SU, UK

## Abstract

Cavitating mesenteric lymph node syndrome (CMLNS) is a rare and poorly understood complication of coeliac disease (CD), with only 37 cases reported in the literature. CD is an immune-mediated enteropathy, with alterations seen in the small bowel architecture on exposure to ingested gluten. Those who fail to respond to a strict gluten-free diet are termed to have refractory coeliac disease (RCD). This is associated with serious complications such as enteropathy-associated T-cell lymphoma (EATL). We present the case of a 71-year-old female investigated for weight loss and a palpable intraabdominal mass. Abdominal computed tomographic (CT) scan showed multiple necrotic mesenteric lymph nodes. At operation, multiple cavitating mesenteric lymph nodes, containing milky fluid, were found. An incidental EATL was found at the terminal ileum, which was resected. The patient subsequently tested positive for CD. This is the second case report to document an association between CMLNS and EATL. This paper highlights the varied presentation of CD. In this case, the diagnosis of CD was made retrospectively after the complications were dealt with. This paper is followed by a review of relevant literature.

## 1. Introduction

Cavitating mesenteric lymph node syndrome (CMLNS) is a rare and poorly understood complication of coeliac disease (CD) with only 37 cases reported in the literature and is associated with a very poor prognosis. Some studies estimate a 50% mortality, mainly related to the complications of severe malnutrition, intestinal haemorrhage secondary to ulceration, and overwhelming sepsis as a result of a combination of hyposplenism and malnutrition. CD is an immune-mediated enteropathy, with alterations seen in the small bowel architecture on exposure to ingested gluten. Those who fail to respond to a strict gluten-free diet are termed to have refractory coeliac disease (RCD), which is associated with an increased risk of malignant neoplasms. Lymphoma accounts for approximately 50% of these, and the most common lymphoma is enteropathy-associated T-cell lymphoma (EATL). 

## 2. Case Presentation

A 71-year-old female with a past medical history of bronchiectasis was investigated by respiratory physicians for a lobulated opacity seen at the right lung base on computed tomographic (CT) scan of the thorax. A repeat CT scan, 2 months later, which included the upper abdomen, showed that the lung opacity had virtually resolved. However, several lymph nodes lying along the mesenteric vessels had now become more prominent, the largest measuring 1.7 cm in short axis. These nodes had necrotic low-density centres and peripheral enhancement. Due to concerns that the patient may have lymphoma or intraabdominal malignancy (the patient also reported coincidental weight loss), a CT abdomen and pelvis was performed. This demonstrated a left ovarian mass with intraabdominal lymph nodes consistent with a potential diagnosis of ovarian cancer with lymphatic and omental metastases. There were no radiological or haematological features of hyposplenism. 

A pelvic ultrasound scan (USS) confirmed a cystic mass with a solid component lying within the left adnexa, measuring 33 × 25 × 59 mm. In light of these findings, total abdominal hysterectomy, bilateral salpingo-oophorectomy, and omentectomy were performed by the gynaecologists. However, pathological examination of the resection specimen was normal. 

Six months later, a follow-up USS showed several large intraabdominal lymph nodes with necrotic centres lying along the midline, the largest measuring 53 mm in diameter. An USS-guided percutaneous biopsy of one of these lesions withdrew 20 mls of thick white material. Two further needle passes only withdrew fragments of material, which were later proven to be skeletal muscle and fibroadipose tissue. 

The patient was then referred to the General Surgical service for minilaparotomy and biopsy of mesenteric lymph nodes. Her only clinical symptom continued to be weight loss. At operation, multiple, large cystic masses within the root of the small bowel mesentery were noted, the largest measuring approximately 15 cm in diameter. Due to the multiplicity of these masses and the fear of the devascularisation of the small bowel with attempted removal, a decision was made against this. Instead, one of the cysts was opened up, draining milky-white fluid, and part of the wall of this cystic mass was excised for histological purposes. This revealed a paucicellular wall containing plasma cells, lymphocytes and fibrous tissue, which was devoid of an epithelial lining and was consistent with a cavitating mesenteric lymph node (see Figure 1). 

An incidental localized small bowel tumour was found in the terminal ileum, and this was removed by means of an ileocaecal resection. Pathological examination and immunohistochemistry revealed this to be an enteropathy-associated high grade T-cell lymphoma. The rest of the small bowel mucosa showed changes of gluten-associated enteropathy with a marked increase in intra-epithelial T cells, villous blunting, and expansion of the lamina propria by small lymphocytes, in keeping with CD. The resection margins were clear of tumour.

The patient made an uneventful recovery and was referred for oncological treatment of her lymphoma (chemotherapy). The diagnosis of CD was further confirmed by positive serum endomysial and tissue transglutaminase IgA antibody levels. At 17-month follow-up she remained free of any disease recurrence and asymptomatic with a repeat CT scan showing a reduction in size of the mesenteric lesions.

## 3. Discussion

This is the second case report in the literature to document an association between CMLNS and EATL. It is well recognised that CMLNS is a rare and poorly understood complication of coeliac disease [1–3].

CD is an inflammatory condition, characterised by an intolerance to ingested grain glutens found in wheat, barley, and rye. If those with the condition are exposed to the gliadin protein component of gluten this leads to an inflammatory process that damages the small bowel mucosa and ultimately leads to malabsorption [4, 5]. CD is multifactorial with an interplay between the trigger; an environmental factor, gluten and genetic susceptibility [5]. The clinical presentation of CD is wide ranging, both in type and severity. Symptoms include abdominal pain, weight loss, and diarrhoea. 

At one end of the spectrum are patients who fail to respond to a strict gluten-free diet, and this is termed refractory coeliac disease (RCD). As well as persisting symptoms, they are at risk of life, threatening complications, which include the development of intestinal T-cell lymphoma and severe malnutrition. At the other end of the spectrum, patients can be almost asymptomatic. There are a number of extraintestinal manifestations that are commonly associated with CD [6, 7].

The pathogenesis of CMLNS is unknown. Characteristically, patients present with refractory symptoms of CD—weight loss, diarrhoea, and fatigue. Clinical signs of hyposplenism may be evident with increased susceptibility to infection. Peripheral blood smear often shows target cells and Howell-Jolly bodies. Villous atrophy is almost always present at small bowel biopsy, as in our case [1, 8]. Multiple cystic masses containing milky creamy fluid are typically present along the jejunoileal mesentery. Typical histopathological examination of these masses shows a pseudocystic, atrophic lymph node with a central cavity containing chylous fluid, surrounded by thin rim of fibrous material and with no evidence of infection or malignancy. These changes are confined to the mesenteric nodal chain [1–3].

Classic CT and sonography findings of CMLNS have been described. Masses are variable in size, ranging from 2 to 8 cm in diameter, and the majority are multiple lesions [1]. On CT, these cystic masses have central low attenuation with a thin enhancing rim. The central material may be fluid or fat. It is possible to see fat-fluid levels on CT, and it is this feature that is thought to be unique to cavitating mesenteric lymph nodes associated with CD. Magnetic resonance imaging (MRI) has been used as a tool to aid diagnosis, as it is able to clearly demonstrate the fat-fluid levels [9]. Sonographically the mesenteric masses are cystic in appearance. Another feature of CMLNS found on radiological investigation is splenic atrophy [2] but this was not present in our patient.

 The pathogenesis of CMLNS is unclear but several theories have been proposed. The small bowel is drained by mesenteric lymphatics termed lacteals, which absorb and transfer emulsified dietary fat via specialised lymph called chyle. It is these specialised mesenteric lymphatics that undergo cystic changes and cavitate. These changes are unique to the mesenteric nodal chain supplying the small bowel. It has been hypothesised that this may be attributed to excessive antigenic exposure of the immune system as a result of damaged intestinal mucosa. This then leads to depletion of cellular lymphoid tissue in the nodes and spleen. Alternatively, the cystic changes may actually reflect necrosis of the mesenteric nodes triggered either by a localised immune-mediated complement cascade and intravascular coagulation, or by trauma [2, 8].

Management of CMLNS is complex. There is no convincing evidence to suggest that regression of the cysts occurs on a strict gluten-free diet as long-term follow-up is limited [3]. Simple aspiration is not recommended due to the high risks of recurrence and infection. Cyst recurrence following surgical resection is unclear. However, surgery has primarily been used for treatment of refractory intestinal ulceration [2, 10].

CMLNS has been associated with a very poor prognosis. Some studies estimate a 50% mortality, mainly related to the complications of severe malnutrition, intestinal haemorrhage secondary to ulceration, overwhelming sepsis as a result of a combination of hyposplenism, and malnutrition [1, 8, 11].

There is an increased risk of malignant neoplasms in patients with CD, the incidence of which is reported as high as 14% [12]. Lymphoma accounts for approximately 50% of these malignant neoplasms, and the most common lymphoma is EATL of the small intestine, which accounts for 85%–90% [13]. Nonlymphomatous malignancies of the gastrointestinal tract account for 24% of all malignancies reported in patients with CD, with adenocarcinoma of the small intestine accounting for 7% of these [12, 14].

EATL is a rare, aggressive malignancy with an incidence of less than 1% of all Non-Hodgkin's lymphoma [15]. Whether it occurs de novo or results from long-term untreated RCD, it has a poor prognosis. The largest reported series in the literature showed 1-year survival of only 31% and 5-year survival of 11% [16]. The histological appearance of EATL is characterised by malignant cells that are highly pleomorphic, with various, abnormal, multinucleated forms [12]. There is no clear consensus regarding the relationship between malabsorption and malignant intestinal lymphoma. Several theories have been postulated: malabsorption predisposes to the development of lymphoma, lymphoma is present throughout the clinical course, or a preneoplastic lesion is responsible for the malabsorption [17, 18]. 

There is a lack of recent data on symptom duration of CD prior to a diagnosis of EATL. In 1967, Harris et al. [13] suggested that this time frame was 21.2 years. However, studies from the 1970s and 1980s suggested that this interval was between 3–5 years, and that, in the newly diagnosed coeliac patient over the age of 50 years, 1 in 10 developed EATL within 4 years of diagnosis of CD [14, 19]. A more recent review of a single centre's data in 2000 identified only 29% of patients with EATL as having had a biopsy-confirmed diagnosis of CD previously [20]. The poor prognosis of patients with EATL is, in part, due to the frequently disseminated nature of the disease at diagnosis and the malnourished condition of the patients. Furthermore, chemotherapy often compounds the depleted nutritional status of the patients, as well as causing treatment-related complications such as gastrointestinal perforation or bleeding [21]. 

This case highlights the complex presentation of CD, especially when it presents with its more serious complications of CMLNS and EATL, and the diagnosis can be challenging. Expedient diagnosis will probably help to decrease the morbidity and mortality associated with untreated coeliac disease.

## Figures and Tables

**Figure 1:**